# Seahawk: moving beyond HTML in Web-based bioinformatics analysis

**DOI:** 10.1186/1471-2105-8-208

**Published:** 2007-06-18

**Authors:** Paul MK Gordon, Christoph W Sensen

**Affiliations:** 1 University of Calgary, Faculty of Medicine, Sun Center of Excellence for Visual Genomics, 3330 Hospital Drive NW, Calgary, AB, T2N 4N1, Canada

## Abstract

**Background:**

Traditional HTML interfaces for input to and output from Bioinformatics analysis on the Web are highly variable in style, content and data formats. Combining multiple analyses can therfore be an onerous task for biologists. Semantic Web Services allow automated discovery of conceptual links between remote data analysis servers. A shared data ontology and service discovery/execution framework is particularly attractive in Bioinformatics, where data and services are often both disparate and distributed. Instead of biologists copying, pasting and reformatting data between various Web sites, Semantic Web Service protocols such as MOBY-S hold out the promise of seamlessly integrating multi-step analysis.

**Results:**

We have developed a program (Seahawk) that allows biologists to intuitively and seamlessly chain together Web Services using a data-centric, rather than the customary service-centric approach. The approach is illustrated with a ferredoxin mutation analysis. Seahawk concentrates on lowering entry barriers for biologists: no prior knowledge of the data ontology, or relevant services is required. In stark contrast to other MOBY-S clients, in Seahawk users simply load Web pages and text files they already work with. Underlying the familiar Web-browser interaction is an XML data engine based on extensible XSLT style sheets, regular expressions, and XPath statements which import existing user data into the MOBY-S format.

**Conclusion:**

As an easily accessible applet, Seahawk moves beyond standard Web browser interaction, providing mechanisms for the biologist to concentrate on the analytical task rather than on the technical details of data formats and Web forms. As the MOBY-S protocol nears a 1.0 specification, we expect more biologists to adopt these new semantic-oriented ways of doing Web-based analysis, which empower them to do more complicated, *ad hoc *analysis workflow creation without the assistance of a programmer.

## Background

### The MOBY-S protocol

**Figure 1:**
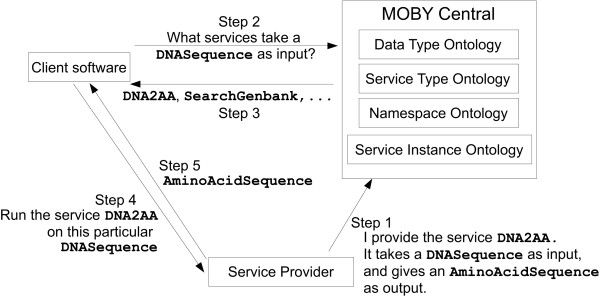
**The MOBY protocol**. Example service registration, discovery and invocation using the MOBY-S protocol. The three actors are the client, MOBY Central, and the service provider. Service registration is "pushed" by the service provider, while service discovery and invocation are "pushed" by the client.

The MOBY-S Protocol [1] has been created by a community of Bioinformatics developers wishing to simplify Web-based analysis. Compatibility of services from different providers is achieved primarily by two means: 1) the ability to programmatically access analysis services (Web Services), and 2) common object representation (common semantics). The former is achieved by using WSDL-based technologies [2] and a service registry (a.k.a. MOBY Central), and the latter by creating ontologies. Figure 1 illustrates the key components of the MOBY-S system.

A key aspect to chaining together services is the ability to directly use output from one service as input to another. In the past, in order to achieve data compatibility between programs, developers would modify existing analysis software and repackage it, or develop completely new programs suites. The most prominent examples of these two approaches were the command-line suites GCG [3] and EMBOSS [4] respectively. In a Semantic Web approach, MOBY-S defines a centralized, world-writable data-type ontology to promote a comprehensive common semantic for biological data. Actual data instances passed on the Web have a standardized XML representation. Several graphical utility programs exist [5] to allow developers to easily browse and edit the service and data-type ontologies, and to register new services. The four MOBY ontologies (see Figure 1) are represented using a combination of OWL and RDF (see [6]), the foundations for the W3C's vision of the Semantic Web. For processing simplicity MOBY uses only a small subset of OWL's expressive power.

### MOBY-S clients and their audiences

MOBY-S, as it approaches a stable 1.0 specification, has the potential to unify analysis in ways other Semantic Web efforts in the Life Sciences to date have not [7]. A large amount of effort thus is being spent in making accessible clients. Currently, there are at least 10 different MOBY-S client programs. They serve a diverse range of niche audiences, from programmers through to average computer users. Programs can be subdivided into three categories based on user skill they assume, from most to least:

• Do construction of a workflow before data instances can be created (visual programming)

• Dynamically build service options based on an entered data instance (standalone browsers)

• Execute MOBY-S Services from within another application (embedded browsers)

In the category of visual programming tools are Taverna [8] and REMORA [9]. With its MOBY-S plug-in [10], Taverna is a Java application which allows the user to build MOBY-S workflows, and then execute the workflows on data loaded from a file, or entered manually. The development of workflows requires a degree of patience and visual programming skills, as the user is not logically guided from one action to the next. Taverna's popularity stems not from simplicity of use, but from its flexibility, robustness, and support for invoking virtually any WSDL-describe Web Service. Taverna provides the ability to execute the workflow over large lists of input, making it ideal for "power-users" who want to process large datasets, but lack traditional programming skills. Settings, such as the time between successive calls to a service, can be set to prevent overloading service providers. REMORA, on the other hand, is HTML-based and acts somewhat more like a browser than Taverna: services are added sequentially, with a list of valid service options presented automatically for every input/output in turn. The user selects the MOBY-S data ontology type and namespace from a list, and object details are filled in after the workflow construction is complete. The workflow is executed, and the user is notified by e-mail. In the results page, each octagonal shape in the workflow is hyperlinked to a simple HTML representation of the data at that stage.

In the category of standalone browsers, from most extensive to simplest user interface, are Dashboard [11], MOWserv [12], Ahab [13] and Gbrowse_moby [14]. Dashboard is a Java application to help MOBY-S service providers register and deploy their services. It includes an interface to create and display MOBY-S Objects, primarily for service testing purposes. Dashboard is a developer-centric interface, as it exposes many of the details of the MOBY-S protocol (including the underlying XML), is service-oriented, and does not choreograph multiple chained invocations. MOWserv is an HTML-based browser where users select data-types from the MOBY-S Data Type Ontology, then fill in the required fields in a form. They proceed to the "Objects" tab, and click on the data to display a list of available services. Service executions are performed asynchronously: they are stored in the "Tasks" tab, and the user checks the status of submitted jobs periodically. While MOWServ provides significant guidance to a user, a drawback of the task queue and tab organization is that the analysis has neither a direct workflow representation for programmers, nor does it sequentially invoke a chain of services as a biologists might expect.

Turning to more biologist-oriented clients, Ahab is an HTML-interface, where available services are shown in a hierarchy and interactive tips about namespaces, data-types and services are displayed. Data entry is simplified by Ahab's exclusive use of basic MOBY-S Objects (database namespace + ID only) to seed the analysis, even if it does somewhat limit the type of analysis available. It also provides an intuitive service-selection hierarchy and logical service chaining. Unfortunately, both the directed graph (default) and text views are cluttered by data structures and relationships only intelligible to those familiar with MOBY-S's RDF [15] technical details. Compare this with Gbrowse_moby, the original MOBY-S client: it provides the simplest interface of all the clients, using hyperlinks on data to chain services together in a Web browser. Like Ahab, it is restricted to basic object input, but unlike Ahab does not display any ontology hierarchies. The textual and sometimes graphical representation of Gbrowse_moby's output is both succinct and sufficient for the vast majority of Bioinformatics data (e.g. sequences, their alignments and annotations).

In the embedded browser category, several applications directly use the Java, Python or Perl MOBY-S libraries to find and/or execute MOBY-S Services. They internally create MOBY-S XML object representations for submission, and parse the service results back into some native display of the application. Such applications include BioTrawler [16], which visualizes protein interaction networks, and both BioFloWeb and AtiDB Client, which implicitly use the European Plant Network's MOBY-S services [17]. Because the use of MOBY-S is programmatic, these types of applications do not have data-type and service selection interfaces, nor a MOBY-specific display interface. An exception to this rule is Seahawk: it is a standalone browser, but it can be easily embedded in existing Java applications as a pop-up menu, as in the genome browser Bluejay [18]. This functionality is described in more detail in the Methods section.

## Implementation

### Defining the biologist's needs

Each of the clients described above serves a niche user type, but how can we get even more biologists to adopt Semantic Web Services? Based on the strengths and weaknesses of those client, a need was identified for an improved way for biologists to access MOBY-S services. The salient observations about existing software are:

• All of the interfaces either accept only simple objects (namespace and id, in Gbrowse), or require a user to build composite objects piece-by-piece. This somewhat limits the type of analysis possible in the former case, and requires an intimate knowledge of MOBY-S's ontologies and data structures in the latter.

• All of the interfaces require a user to go to a particular Web page (or a CVS download in the case of Dashboard), and manually input data. This manual effort requires the user to already be familiar MOBY-S's object and namespace ontologies, in order to formulate the data. Users are also required to break away from their other applications to use MOBY-S.

• As most data in Bioinformatics is textually represented, hypertext (HTML) interfaces are the most natural fit for displaying data (and hence its popularity as a presentation medium for MOBY client software so far). While HTML pages are easy for biologists to work with, for any given hypertext client described here, there are different pages associated with 1) MOBY data input, 2) MOBY data display and 3) MOBY service selection. Users must constantly flip between service and data page "modes" to chain together an analysis.

• Using visual programming tools creates reuseable workflows, but they are relatively difficult for biologists to use, compared to browsing in the other clients.

To address these issues, Seahawk attempts to provide:

• Creating Input: The ability to modify and extend the automated linking of existing Bioinformatics data to MOBY-S Service (and seed analysis with composite MOBY-S Objects).

• Embedding: The ability to easily link MOBY-S Services into existing Bioinformatics software.

• Browser Interface: More interactivity versus the HTML interfaces previously described, and improved usability versus the visual programming tools for the most common types of analysis.

• Output: The ability to create workflows more easily than the visual programming clients.

### Creating input

With the exception of MOWServ (where objects with many fields can be built manually), the HTML-based interfaces for MOBY-S are all seeded with basic MOBY-S Objects having a (namespace, id) tuple. This assumes first of all that the user is accessing a piece of data already in existence in a database, and that the database is connected to MOBY. Unfortunately, both assumptions are often false. Users may be interested in analyzing a new sequence they have just elucidated in the lab, have yet to submit to a public database (pre-publication), or any one of many other reasons. Even if they are accessing a published piece of data, it is quite possible that the database they are using has not yet been "hooked into" MOBY-S by any developers.

The vast majority of Bioinformatics data is available as formatted text, or HTML through Web sites. Seahawk accommodates importing as many file formats as possible for "seeding" the analysis, namely:

1. Plain text (e.g. a FastA formatted file)

2. HTML (e.g. an NCBI Entrez Web Page)

3. Rich Text (e.g. a conference proceedings)

4. MOBY-S object XML representation (e.g. output from a MOBY-S Service)

Data can be loaded from file:, ftp:, or http: URLs using the disk icon in Seahawk, or by simply using cut and paste, or drag and drop facilities of the operating system. This input flexibility means that the user's existing desktop files, Web links, and highlighted parts of Web pages (e.g. an NCBI Genbank entry page) can be directly manipulated and used as Seahawk analysis input.

**Figure 2:**
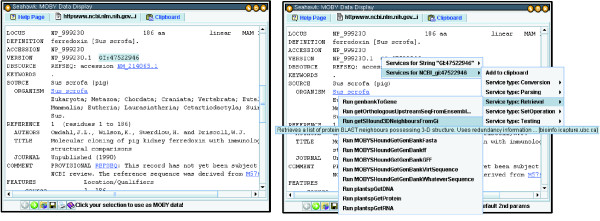
**Seahawk's data-creation-by-highlighting capability**. Left: Selection of a GI number within an NCBI Entrez Web page for pig ferredoxin (note hint in status bar). Right: MOBY-S Services associated with GI number input (as registered in MOBY Central), listed in a hierarchical popup menu.

As we have already seen, MOBY-S Objects and their subcomponents can be selected using the hyperlinks available in their HTML display. Seahawk imports several non-XML data formats, and a user may want to analyze only subsections of a MOBY-S Object's character string representation. To accomodate such partial data usage, it was important to provide a text-selection facility in Seahawk. In Seahawk, a user can highlight any arbitrary text in the display by a mouse drag, and have the text automatically converted to MOBY-S Objects for service execution, as illustrated in Figure 2.

There are 3 main mechanisms that can create MOBY-S Object instances from selected character strings in Seahawk:

1. Highlighted text is automatically turned into a MOBY-S String

2. Seahawk will create a MOBY-S DNASequence, RNASequence or AminoAcidSequence if 95% of the text characters are valid for that sequence type (the 5% exception is meant to deal with formatting characters, such as position numbers in the leading columns of GenBank records). The invalid characters are stripped from the data.

3. The text is tested against a set of regular expression rules

The regular expression and XPath rules are specified in a special rules file described in the Methods section. The three sequence object types described above are the only ontology terms hardcoded into Bluejay, but could be overridden by new regex rules if these terms change.

### Embedding Seahawk in other applications

**Figure 3:**
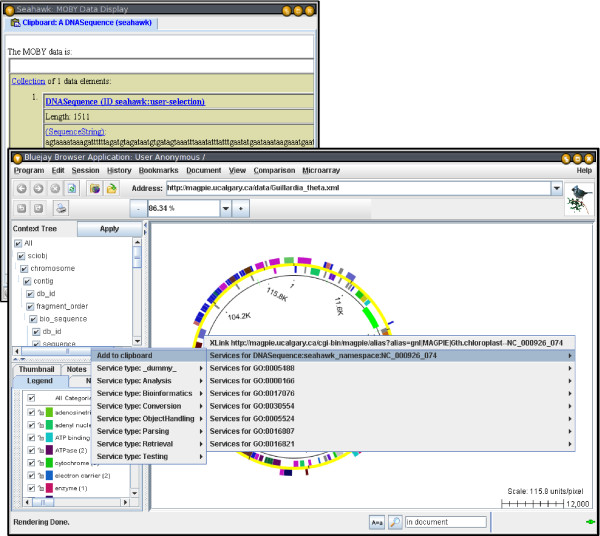
**Seahawk embedded in another application**. DNASequence constructed from Bluejay selection added to Web Service pop-up menu. The DNASequence is added to the Seahawk Clipboard (top-left), an example of transferring a composite MOBY-S Object to Seahawk from another application. The Bluejay user is exposed to Web Services without leaving the main application.

When Seahawk is used as a helper application, the main application may programmatically add data to Seahawk's clipboard. In Figure 3, the Bluejay genome browser application [18] creates a DNASequence object (using the Java MOBY-S libraries) based on the glyph clicked in the display. This data can then be passed on to the clipboard using the pop-up menu. Applications can therefore seed MOBY-S analysis with arbitrarily composite MOBY-S Objects they construct themselves. More information on application integration can be found in the Methods section.

### Using and improving the Web browser paradigm

**Figure 4:**
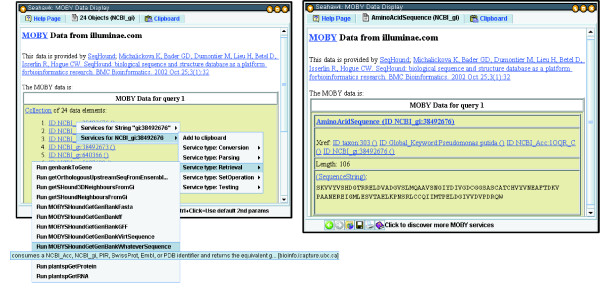
**Seahawk's interface: tabs, hyperlinks and nested popup menus**. In the compact tabbed Web-browser interface of Seahawk, navigation and other browser functions are found at the bottom of the screen, and hyperlinks abound in the document. **Left**: The 28 results of the getSHound3DNeighboursFromGi service invocation from Figure 2. The pop-up menu appears after clicking one of the NCBI_gi hyperlinks, and presents services that take an object in the NCBI_gi namespace as input. **Right**: Results of the Genbank record retrieval service for the selected NCBI gi number. Note that service hyperlinks appear for crossreference and sequence subcomponents of the composite object AminoAcidSequence.

In order to make the user experience as intuitive as possible, Seahawk uses the tabbed-Web browser user interface design, which will be familiar to most potential users. The tabbed interface (Figure 4) allows the user to branch off different investigation paths by launching services in new tabs. Within a tab, the linking from one service output to the next is sequential, and stored in a history for back-and-forward functionality (via arrows on the bottom tool bar).

This interface makes the inquiry task and data-centric, rather than service-centric: the users ask themselves what they should do next with the data, not what data do they need to run a particular service. Seahawk improves upon the other HTML-based MOBY-S clients by avoiding constant browser-page changes. This is accomplished by displaying service choices and input parameters as pop-up menus and dialog windows respectively.

**Figure 5:**
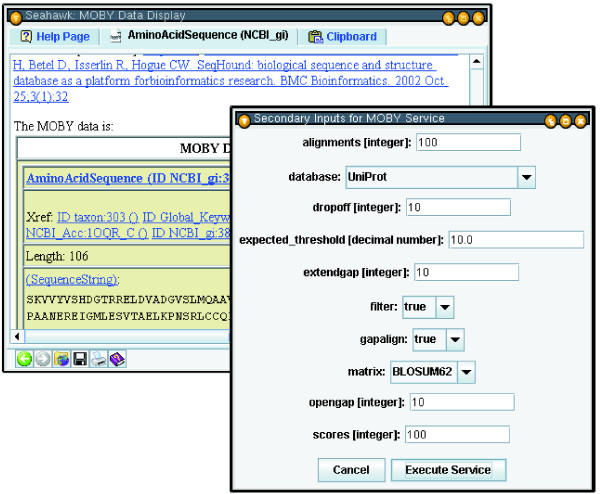
**Example Seahawk service parameter dialog**. Seahawk's auto-generated secondary input interface for a BLAST Web Service. Enumeration parameters are represented as drop-down boxes, strings and integers as text fields.

Providing service choices via pop-up menus has several advantages. First, all browser window real estate can be dedicated to displaying the scientist's data of interest. Underlining hyperlinks provides a familiar yet unobtrusive visual clue for document navigation. Second, hyperlinks' negligible space requirement allows Seahawk to specify links for all subcomponents of a MOBY-S data document, making data decomposition intuitive. For example, in Figure 5, a service options hyperlink is available for the AminoAcidSequence object, but also for its crossreference, and the data member SequenceString (which will show the MOBY-S Services accepting the String data-type as input). Third, when many service options are available, pop-up submenus can hierarchically organize the services and ease the user's navigation of the options. Services may also designate auxiliary parameters that control the service's behaviour; these are referred to as *secondary inputs*. All secondary inputs must have a default value, in order to make an uninformed user's submission process easier. They may also have a minimum and maximum range if numeric (integer or floating point), or an enumeration of choices (if a string). If a service with secondary parameters is invoked in Seahawk, a non-modal dialog box is dynamically generated and displayed to allow the user to change these secondary parameters, as shown in Figure 5.

By launching a dialog rather than loading an HTML-form in the browser, Seahawk maintains its browser-display-equals-service-results philosophy. Secondary input does not enter the browser history, and because it is non-modal, the user may defer service execution while they explore other analysis choices. The user can avoid the dialog altogether, and simply use the default values, by holding down the Control key while selecting the service. This feature makes service navigation even simpler.

While Seahawk has no facility to edit the MOBY-S Objects displayed (as the edits might break logical or biological constraints of the object), the user may put object collections, individual objects, or object members on the clipboard using the "Add to clipboard" option available from every service selection menu. The clipboard allows the user to pick salient data from any step of the analysis for use later on, providing a way to arbitrarily combine information from multiple services (pages) or analysis branches (tabs).

**Figure 6:**
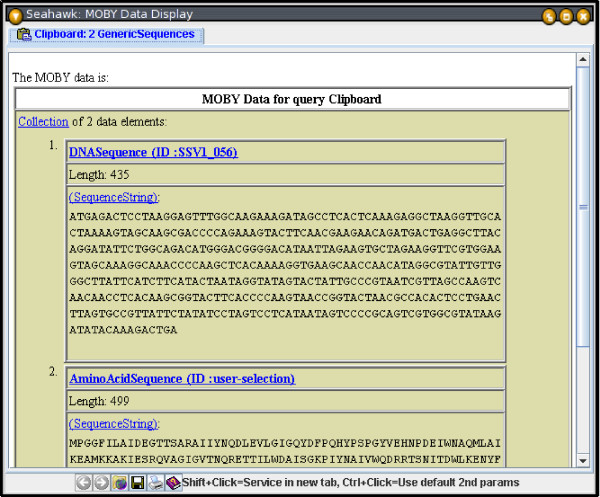
**Seahawk's intelligent clipboard**. Seahawk clipboard with a DNASequence and an AminoAcidSequence. By checking the data-type ontology, Seahawk infers that it has a collection of 2 GenericSequences, their nearest common ancestor (as seen in the tab title).

Individual clipboard items, or the whole clipboard, can also be cleared. The multi-item clipboard has no equivalent in any major Web browser, but is a familiar concept to most computer users. The MOBY-S Objects on the clipboard can be used individually to launch services, or they may be used as a MOBY-S Object Collection for input to a service. The data-type of the MOBY-S Object Collection is determined by finding the nearest common ancestor of all objects in the data-type ontology, as demonstrated in Figure 6. Dynamic collection data typing is an example of domain-specific semantics automatically being applied to the application interface. The "downgrading" of object collection data types does not affect the individual members of the collection: members are still passed to the service with all their fields (not just the common ones) intact, in case the service can use them constructively.

### Output

**Figure 7:**
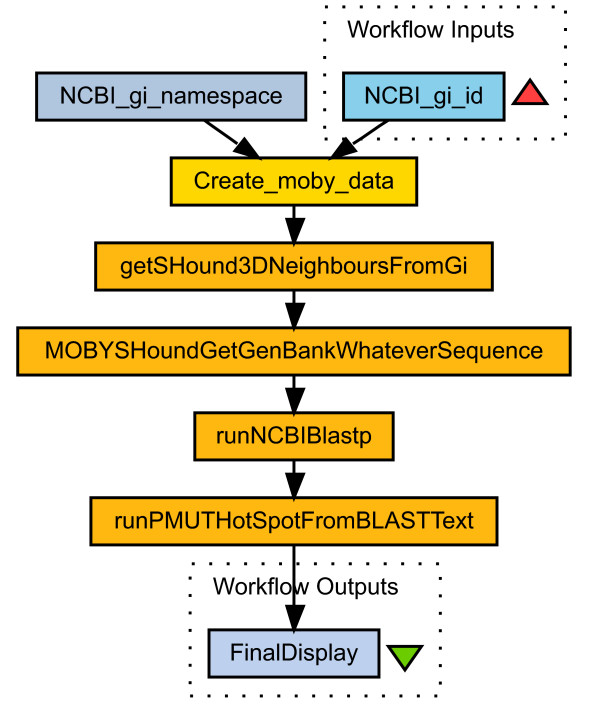
**Seahawk as a workflow generator**. Abstract workflow derived by Seahawk from the browsing done in Figures 2, 4, and 5, followed by a PMUT [23] analysis of the BLAST results.

The user has several choices on how to save data from Seahawk. First, the MOBY-S Objects on-screen can be saved directly into an XML file. This allows a user to resume their inquiry at a later date by reloading the saved document. The on-screen data can also be saved in HTML format, for sharing with colleagues, import into a word processor, etc. Finally, the browsing history for a particular Seahawk tab can be saved as a (linear) workflow. The browsing session performed in this paper's figures can be abstracted into a workflow as in Figure 7.

**Figure 8:**
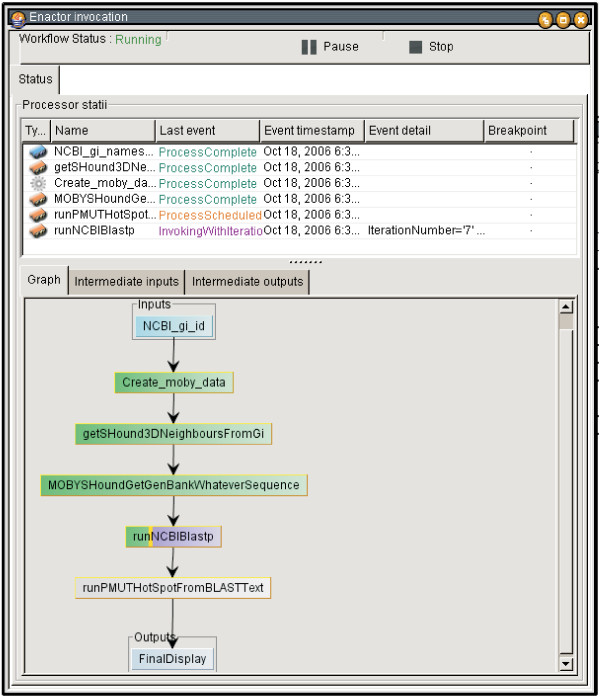
**Seahawk workflow being executed in Taverna**. When using Taverna, the object collection returned by the first service causes an implicit for loop on the remaining servicse: all 28 NCBI_gis (corresponding to ferredoxin-like proteins) will have their sequence retrieved, BLASTed, and analyzed for mutation sensitivity. The user's simple example browsing has been used to generate a much larger dataset.

The workflow is encoded by Seahawk code as a SCUFL XML file that can be loaded into Taverna and re-enacted at any point in the future on large lists of input data (see Figure 8). Building the example workflow directly in Taverna requires at least 24 user actions, versus 8 actions (including 3 hyperlink clicks) in Seahawk. This workflow export feature of Seahawk constitutes a basic programming-by-example functionality. The Gbrowse client has also just recently implemented a simple workflow exporter. We hope that use of workflows becomes more widespread as they becomes more readily available, and programs such as Seahawk can become a bridge for users into auditable execution enviroments such as Taverna. The ability to technically document the analysis process in a workflow addresses two issues in Bioinformatics today: proper citation [19] and reproducibility of the results.

## Results

The Seahawk software described here consists of approximately 15,000 lines of Java code. Seahawk also uses some existing Java code from the BioMOBY public code repository (hosted at cvs.open-bio.org). A theme throughout the implementation of Seahawk is to lower the entry barrier to the Semantic Web for users *and *developers. This is achieved in practice via the use of several XML technologies (DOM, XSLT, XPath [20]) so that customization of Seahawk can be done without Java coding. The use of declarative programming (e.g. XSLT, XPath) for customization are numerous in the context of semantic data manipulation (paper in preparation), such as modularity, security, and low developer buy-in. The reader is directed to the BioMOBY Website [21] for complete, concrete examples of the customization methodologies described in this section.

### Data display

Seahawk converts raw MOBY-S XML returned from services into HTML suitable for display in a javax. swing.JEditorPane. This conversion is done using an XSLT processor (discovered at run-time using the JAX-T API), and an XSLT style sheet. The conventions used for the transformation and subsequent display are:

• Seahawk will interpret any URL with a numeric XPointer (e.g. file:///foo.xml#/1/2/3/4/4) as a link to part of a MOBY-S XML document, and hence will automatically provide MOBY-S Service links when clicked, by parsing the data at that XPointer

• Hyperlinks of the form http://moby/namespace value?id=id_value will be used by Seahawk to construct basic MOBY-S database identifier objects, for linking out to relevant MOBY-S Services.

• All other hyperlinks will be launched into an external browser (e.g. Firefox or Internet Explorer)

The underlying XML representation of the semantic data is always retained, even if the HTML interface is changed via new style sheets. This means that there are no potential risk of Seahawk inadvertently changing the data as it brokers the passage of messages from one MOBY-S Service to the next.

### Creating MOBY-S data With a rules file

Seahawk provides the ability to map unstructured text, or any XML document data, into MOBY-S semantic data via a rules file. The rule set can be easily augmented as developers adopt Seahawk for their data. The rules file is written in XML, with a base element called mappings, which holds any number of object children.

The object elements represent templates for MOBY-S Objects to construct. Tags nested inside the object tag populate the various MOBY-S Object field instances. The simplest MOBY-S Objects have just a (namespace, identifier) attribute pair. For example, the example in Table 1 defines one regular expression rule, to build a MOBY-S Object in the NCBI global identifier namespace ("NCBI_gi" in MOBY's Namespace Ontology).

**Table 1:** Seahawk data rule format: regular expressions. Complete rules file, containing one regular expression rule for creating a basic MOBY-S NCBI Global Identifier record from a string, such as "gi"122354" or "GI:636353". Captured groups from the regex can be used to populate the MOBY-S Object fields using the standard Perl and Java syntax ($1, $2, etc.).

<?xml version="1.0"?>
<mappings>
<object>
<regex>(?:GI|gi) [:|](\ d+)</regex>
<namespace>
<ns value="NCBI_gi">$1</ns>
</namespace>
</object>
</mappings>

XPath rules are used to build MOBY-S Objects from in-memory XML Document Object Models (DOMs). In contrast to the regular expressions, which pick salient substring from a simple text character sequence, XPath rules search the highly structured data of a DOM. XPath rules consequently are considerably more flexible and powerful (see Table 2 for an example).

**Table 2:** Seahawk data rule format: XPath expressions. XPath-based rule for creating a basic MOBY-S Gene Ontology record from a DOM data source, in this case an AGAVE XML document. The DOM context node for the XPath evaluation is determined by the application.

<prefix value="agave">http://www.bioxml.info/dtd/agave.dtd</prefix>
<!-- Build a MOBY Object in the Gene Ontology namespace-->
<object>
<!-- Find gene elements w/GO classification children-->
<xpath>self::agave:gene//agave:classification [@system='GO']</xpath>
<namespace>
<!-- Find the ID attribute of the above xpath result-->
<ns value="GO">./@id</ns>
</namespace>
</object>

Note that the "./@id" in the namespace rule is another XPath statement. Its context is the results of the xpath rule, and fetches the id attribute of the AGAVE classification element.

### Embedding Seahawk in other applications

To simplify the use of Seahawk as a component inside another program (such as the Bluejay example given earlier), a specialized java.lang.ClassLoader was written. This ClassLoader captures all of the classes required to run Seahawk (determined by running a series of automated tests), and puts them in one JAR file. This minimalist JAR builder allows any developer to include a single JAR file in their program to access Seahawk. It is also used to minimize the applet download size. The file contains just the relevant classes to run Seahawk – such as those from the BioMOBY CVS, and from The Apache Foundation's [22] Axis (SOAP), Xalan (XSLT), Xerces (XML parsing) and XPath packages. Whereas these packages in their totality constitute about 20 MB in JAR files, the minimized package provides a standalone, fully functional MOBY-S Services browser in less than 3 MB.

The definition of XPath rules requires greater skill set than building regular expression rules, and is aimed primarily at developers who will integrate Seahawk into their existing XML-based applications. The XPath rules provide the bridge between the application's data and the MOBY-S data format. As such, an application using a DOM needs very few changes to embed Seahawk's functionality. The code in Table 3 demonstrates how Seahawk is integrated into a Java application.

**Table 3:** Integrating Seahawk into other Java code. Complete Java code required to integrate Seahawk into a DOM-based application. The developer might also want to add rules specific to their application.

import ca.ucalgary.seahawk.util.MobyUtils;
import ca.ucalgary.seahawk.gui.MobyContentGUI;
MobyContentGUI mGUI = MobyUtils.getMobyContentGUI(null);
//Pick W3C DOM node 'contextNode' for rules eval, then...
popup = new JPopupMenu();
//Evaluate Seahawk's XPath rules, from that node
mGUI.addPopupOptions(contextNode, popup, true);//true=async
popup.setVisible(true);

## Discussion

### Improved features

Seahawk improves the user experience over existing MOBY-S clients with two main features: pop-up menus from hyperlinks and clipboard functionality.

Introducing hyperlinked pop-up menus to display service options has several advantages. First, the user is not sent to a new page to select the service (as happens in other clients). Treating services as hyperlinks between input and output data maintains a data-centric browsing experience for end-users. Second, the pop-up menu does not occupy any screen real-estate when not in use, but still provides a detailed (tool-tips) logical (ontology-based) hierarchy when in use. Third, the hyperlinks allow for easy object decomposition because they can be inserted for each object member without affecting the display's readability.

The clipboard helps Seahawk cross over from purely a browser to a browser/editor hybrid. The clipboard acts as a collator to MOBY-S Object Collections, allowing users to combine objects as they see fit. It also allows a user to temporarily keep data from various steps of the analysis, without keeping many tabs open. Individual members of a composite object can be chosen and added to the clipboard too, facilitating MOBY-S Object decomposition. The clipboard, like any tab, can be saved to disk, and reopened in another Seahawk session in the future.

### Novel features

Seahawk introduces three novel features to Web Services clients in general: data-creation-by-highlight, rule-based systems for data mapping, and service-interface-as-component for application integration. Data import and data-creation-by-highlighting together provide an important facility to the biologist: creating MOBY-S Objects with semantic meaning out of plain text. This allows the user to import an array of existing text-based data into a Semantic Web Service system, including the many standard Web resources the user is familiar with already. Such a bridge from the existing Web to the Semantic Web is essential to user adoption. Highlighting is also especially important to biologists because it allows them to easily select subsequences of DNA and protein that they deem biologically meaningful.

The unique regular expression and XPath based rule system for creating MOBY-S Objects improves the user experience both directly and indirectly. In addition to being the mechanism by which highlighting text generates structured data objects, it allows Seahawk to directly "hook into" XML-based third-party applications. Users indirectly benefit too: the rules system allows developers to easily add new data mappings, and hence new analysis possibilities.

The visual simplicity of pop-up menu service selection helps make Seahawk blend in with external applications that use it as a helper component. The focus on making Seahawk a small JAR, with an easy to use API, is meant to encourage the embedding of Seahawk within Java applications. By integrating Seahawk into their existing applications, Bioinformatics developers can provide the power of Semantic Web Services to the end-user without making them go to a separate application, and manually transfer the relevant data.

## Conclusion

Traditionally, Web Services have been oriented towards developers, who predetermined the service to be called, then wrapped the service execution and response within another program. The real key to empowering the biologist is to have domain-specific ontologies that can help the user, rather than the programmer, select appropriate data and analysis options. The MOBY-S system provides such ontologies for Bioinformatics.

Seahawk is a MOBY-S client built on the foundation of the Web-browser interface, familiar to virtually all potential users, not just developers. Seahawk hides all of the underlying implementation details of MOBY-S from the user, lowering the barrier to using Semantic Web Services. Many features of Seahawk can be classified as either "improved" or "new" based on their degree of novelty compared to other Web Services software and especially other MOBY-S clients. To improve the end-user experience, the key on the front-end is the incorporation of UI elements that keep the experience data-centric, treating services as links between data. The key on the back-end is making it as easy as possible to create semantic data from data the user is already familiar with (primarily Web pages and flat-file records), addressed with a novel regular expression/XPath rule system, and application embedding.

Much Bioinformatics analysis happens on the Web because information and resources are scattered amongst many labs. There are three key actors in the Semantic Web for Life Sciences, users (biologists), application developers, and service providers. Seahawk lowers the barriers for user and developer adoption. Adoption of MOBY-S by service providers is gaining momentum as the protocol approaches version 1.0. A critical mass of all three actors will allow us to empower the biologist to seamlessly perform multi-step analysis in this largely Web-based field.

## Availability and requirements

Project name

Seahawk

Project home page



Operating systems

Platform independent

Programming language

Java 1.5 or higher

License

GNU Lesser General Public License (LGPL)

Any restrictions to use by non-academics

None

## Authors' contributions

PG is responsible for all of the coding of Seahawk. PG designed the novel user interaction paradigm of Seahawk, in collaboration with CS. PG wrote the manuscript, with critical review by CS. Both authors read and approved the final manuscript.
